# Scalable Approach to Consumer Wearable Postmarket Surveillance: Development and Validation Study

**DOI:** 10.2196/51171

**Published:** 2024-04-04

**Authors:** Richard M Yoo, Ben T Viggiano, Krishna N Pundi, Jason A Fries, Aydin Zahedivash, Tanya Podchiyska, Natasha Din, Nigam H Shah

**Affiliations:** 1 Department of Medicine, School of Medicine, Stanford University, Stanford, CA, United States; 2 Department of Cardiovascular Medicine, School of Medicine, Stanford University, Stanford, CA, United States; 3 Department of Pediatrics, School of Medicine, Stanford University, Stanford, CA, United States; 4 Veterans Affairs Palo Alto Health Care System, Palo Alto, CA, United States; 5 Clinical Excellence Research Center, School of Medicine, Stanford University, Stanford, CA, United States; 6 Technology and Digital Services, Stanford Health Care, Stanford, CA, United States

**Keywords:** consumer wearable devices, atrial fibrillation, postmarket surveillance, surveillance, monitoring, artificial intelligence, machine learning, natural language processing, NLP, wearable, wearables, labeler, heart, cardiology, arrhythmia, diagnose, diagnosis, labeling, classifier, EHR, electronic health record, electronic health records, consumer, consumers, device, devices, evaluation

## Abstract

**Background:**

With the capability to render prediagnoses, consumer wearables have the potential to affect subsequent diagnoses and the level of care in the health care delivery setting. Despite this, postmarket surveillance of consumer wearables has been hindered by the lack of codified terms in electronic health records (EHRs) to capture wearable use.

**Objective:**

We sought to develop a weak supervision–based approach to demonstrate the feasibility and efficacy of EHR-based postmarket surveillance on consumer wearables that render atrial fibrillation (AF) prediagnoses.

**Methods:**

We applied data programming, where labeling heuristics are expressed as code-based labeling functions, to detect incidents of AF prediagnoses. A labeler model was then derived from the predictions of the labeling functions using the Snorkel framework. The labeler model was applied to clinical notes to probabilistically label them, and the labeled notes were then used as a training set to fine-tune a classifier called Clinical-Longformer. The resulting classifier identified patients with an AF prediagnosis. A retrospective cohort study was conducted, where the baseline characteristics and subsequent care patterns of patients identified by the classifier were compared against those who did not receive a prediagnosis.

**Results:**

The labeler model derived from the labeling functions showed high accuracy (0.92; *F*_1_-score=0.77) on the training set. The classifier trained on the probabilistically labeled notes accurately identified patients with an AF prediagnosis (0.95; *F*_1_-score=0.83). The cohort study conducted using the constructed system carried enough statistical power to verify the key findings of the Apple Heart Study, which enrolled a much larger number of participants, where patients who received a prediagnosis tended to be older, male, and White with higher CHA_2_DS_2_-VASc (congestive heart failure, hypertension, age ≥75 years, diabetes, stroke, vascular disease, age 65-74 years, sex category) scores (*P*<.001). We also made a novel discovery that patients with a prediagnosis were more likely to use anticoagulants (525/1037, 50.63% vs 5936/16,560, 35.85%) and have an eventual AF diagnosis (305/1037, 29.41% vs 262/16,560, 1.58%). At the index diagnosis, the existence of a prediagnosis did not distinguish patients based on clinical characteristics, but did correlate with anticoagulant prescription (*P*=.004 for apixaban and *P*=.01 for rivaroxaban).

**Conclusions:**

Our work establishes the feasibility and efficacy of an EHR-based surveillance system for consumer wearables that render AF prediagnoses. Further work is necessary to generalize these findings for patient populations at other sites.

## Introduction

### Background

Consumer-facing devices such as the Apple Watch [1] and Fitbit [2] now have the capability to notify users with a *prediagnosis* such as atrial fibrillation (AF). As these notifications may incentivize patients to seek follow-up medical care, wearables now have the potential to affect diagnosis rates and initiate cascades of medical care [3,4]. Although these devices undergo premarket validation to obtain Food and Drug Administration (FDA) clearance [5], limited information exists on their postmarket use and clinical utility.

To conduct *postmarket surveillance* on consumer wearables, electronic health records (EHRs) should capture wearable use, in particular those incidents where patients received prediagnosis notifications. However, EHRs are often built around medical diagnosis codes used for billing purposes [6,7], which do not contain terms for describing wearable use. Prescription wearables should have ordering information, but this does not capture how the wearables are used. Therefore, unstructured data such as clinical notes must be parsed to obtain the wearable use information.

Deep learning–based natural language processing (NLP) methods [8-10] have been shown to outperform traditional approaches on clinical note classification tasks [11,12]. However, these deep learning–based classifiers require large, hand-labeled training sets that are costly to generate. For EHR-based postmarket surveillance to be widely implemented, a scalable approach is necessary to reduce the labeling burden.

### Objectives

We aimed to demonstrate the feasibility and efficacy of postmarket surveillance on consumer wearables that render AF prediagnoses. The first aim of this study was to evaluate the efficacy of a weakly supervised approach to heuristically generate labels for a training set. A *labeler model* derived from programmatically expressed heuristics probabilistically assigns labels to clinical notes regarding whether the note contains a mention of the patient receiving a prediagnosis from a wearable. The second aim was to evaluate the performance of a *classifier* fine-tuned on the training set labeled by the labeler model, which identifies mentions of an AF prediagnosis in a note. The third aim was to summarize the clinical characteristics of patients identified by the classifier and compare them to patients who were not alerted to a prediagnosis.

## Methods

### Cohort Identification

We used the Stanford Medicine Research Data Repository (STARR) data set [13], which contains EHR-derived records from the inpatient, outpatient, and emergency department visits at Stanford Health Care and the Lucile Packard Children’s Hospital. We retrieved all clinical notes from the STARR data set that contain a mention of a wearable device (Textbox 1), resulting in 86,260 notes from 34,329 unique patients. Following the FDA guidance for pertinent cardiovascular algorithms [5], we excluded patients younger than 22 years of age when the note was written, leaving 78,323 notes from 30,133 unique patients. We further limited the data set to notes written on or after January 1, 2019, since the first consumer-facing AF detection feature became available in December 2018 [14]. The resulting cohort comprised 56,924 notes from 21,332 unique patients.

Textbox 1. **Search terms for wearable devices.**

Apple watch, iwatch, applewatch, fitbit, fit bit, fit-bit, galaxy watch, samsung watch, google watch, kardia, alivecor, alive cor, wearable, smart watch, and smartwatch

To evaluate the performance of the labeler model and the classifier, we constructed a test set by manually labeling 600 notes. Specifically, we randomly selected 600 unique patients and then selected 1 note for each patient that contained *action terms* (Textbox 2) in the vicinity (30 characters) of a wearable mention. This was to filter out nonrelevant wearable descriptions (eg, boilerplate texts recommending the use of wearables during meditation), so that resulting notes are enriched with relevant use cases.

Textbox 2. **Action terms used to enrich sample relevance.**

Alert, notify, warn, observe, identify, detect, note, record, capture, show, report, give, alarm, register, read, tell, have, had, see, saw, receive, get, got, notice, check, and confirm

These notes were then labeled independently by 2 data scientists, and differences were adjudicated by 2 physicians. A clinical note was labeled as positive when the patient received an automated AF notification from the wearable, or when the patient initiated an on-demand measurement (eg, electrocardiogram strip) that resulted in an AF prediagnosis. There were no instances where the 2 physicians disagreed on the label. The resulting test set contained 105 positive notes (prevalence=0.18).

In addition to the test set, we prepared a development set of 600 notes that was used to aid the development of the labeler model. This set was manually labeled by a single data scientist, using a labeling guideline (Multimedia Appendix 1) that was developed as part of the test set generation. The development set contained 100 positive notes (prevalence=0.17).

### Labeler Model Derivation

**Figure 1.**
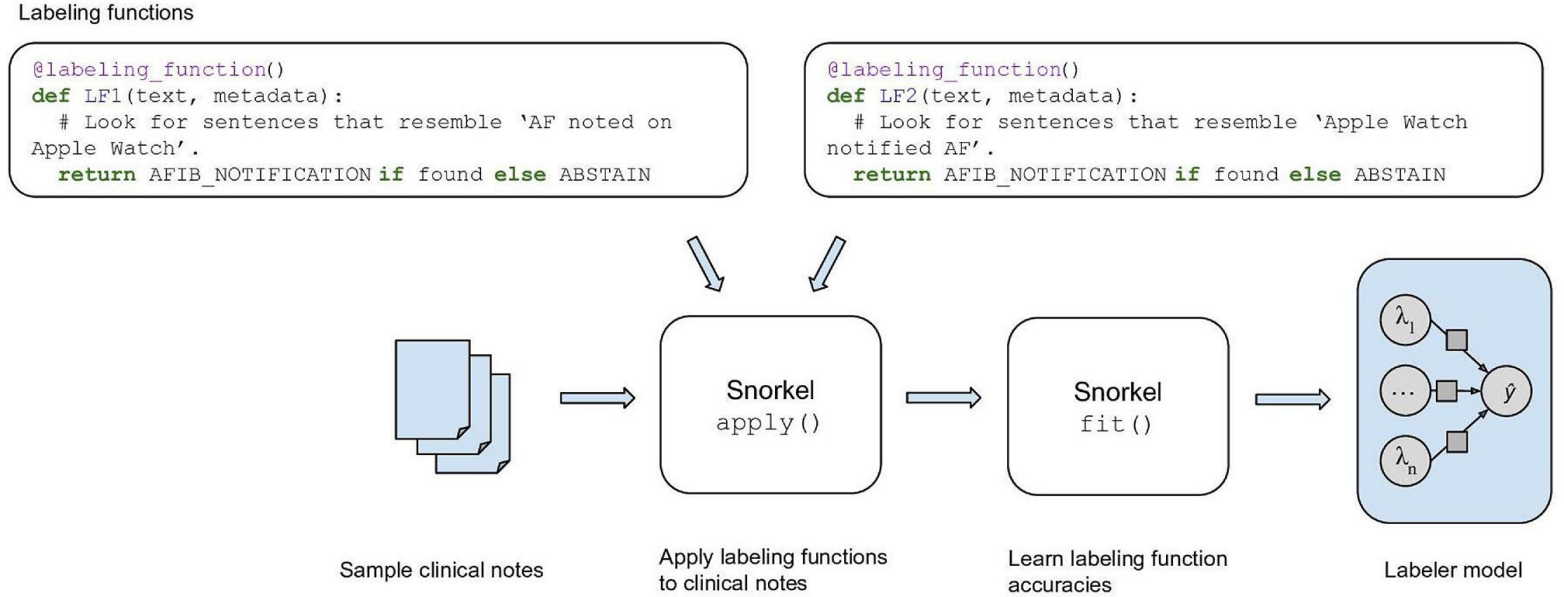
Labeler model generation process. Labeling heuristics were expressed as code-based labeling functions. Snorkel [16] then applied the labeling functions to the sample clinical notes and fit a generative model on the predictions of the labeling functions. The resulting labeler model probabilistically assigns a label to a clinical note based on whether the note mentions the patient receiving an AF prediagnosis from the wearable device. AF: atrial fibrillation.

We then derived a labeler model that used weak supervision to probabilistically assign labels for the training set. Specifically, as shown in Figure 1, we used data programming [15], where labeling heuristics are expressed as code-based *labeling functions*. Using the encoded heuristics, the labeling functions make predictions as to which label a clinical note should be assigned. Predictions from these labeling functions are then combined to develop a generative *labeler model*.

We used the Snorkel framework [16] to implement data programming. A preprocessing framework [17] was applied to our notes to split them into sentences using the spaCy [18] framework, with a specialized tokenizer to recognize terms specific to medical literature. Thus parsed grammatical information was made available to the labeling functions as metadata.

We then used the development set to understand how the AF prediagnosis was described, and we expressed each pattern as a labeling function. The development process was iterative, where the Snorkel framework allowed us to observe the predictive values of the labeling functions on development set records. Each function could then be further optimized to reduce the differences between predictive values and actual labels, leading to overall performance improvement on the development set. Textbox 3 shows all the terms that were identified as denoting AF. Negations were properly handled.

Once developed, we applied the labeling functions on the samples and then instructed Snorkel to fit a generative model on the output. Specifically, we used 10-fold cross-validation on the test set and chose the labeler model with the best *F*_1_-score. This model was then applied to the entire corpus of 56,924 notes to probabilistically assign labels.

Textbox 3. **Terms denoting atrial fibrillation.**

Af, afib, a-fib, a.fib, arrhythmia, paf, atrial fibrillation, a. fib, a fib, atrial fib, atrial arrhythmia, irregular heartbeat, irregular hr, irregular rhythm, irregular pulse, irreg hr, irregular heart beat, irregular heart rhythm, irregular heart rate, irreg heart rhythm, irreg heart beat, irreg heart rate, abnormal ekg rhythm, paroxysmal atrial fibrillation, a. fib, a - fib, pafib, abnormal heart rhythm, abnormal rhythm, abnormal HR, and arrhythmia

### Classifier Fine-Tuning

**Figure 2.**
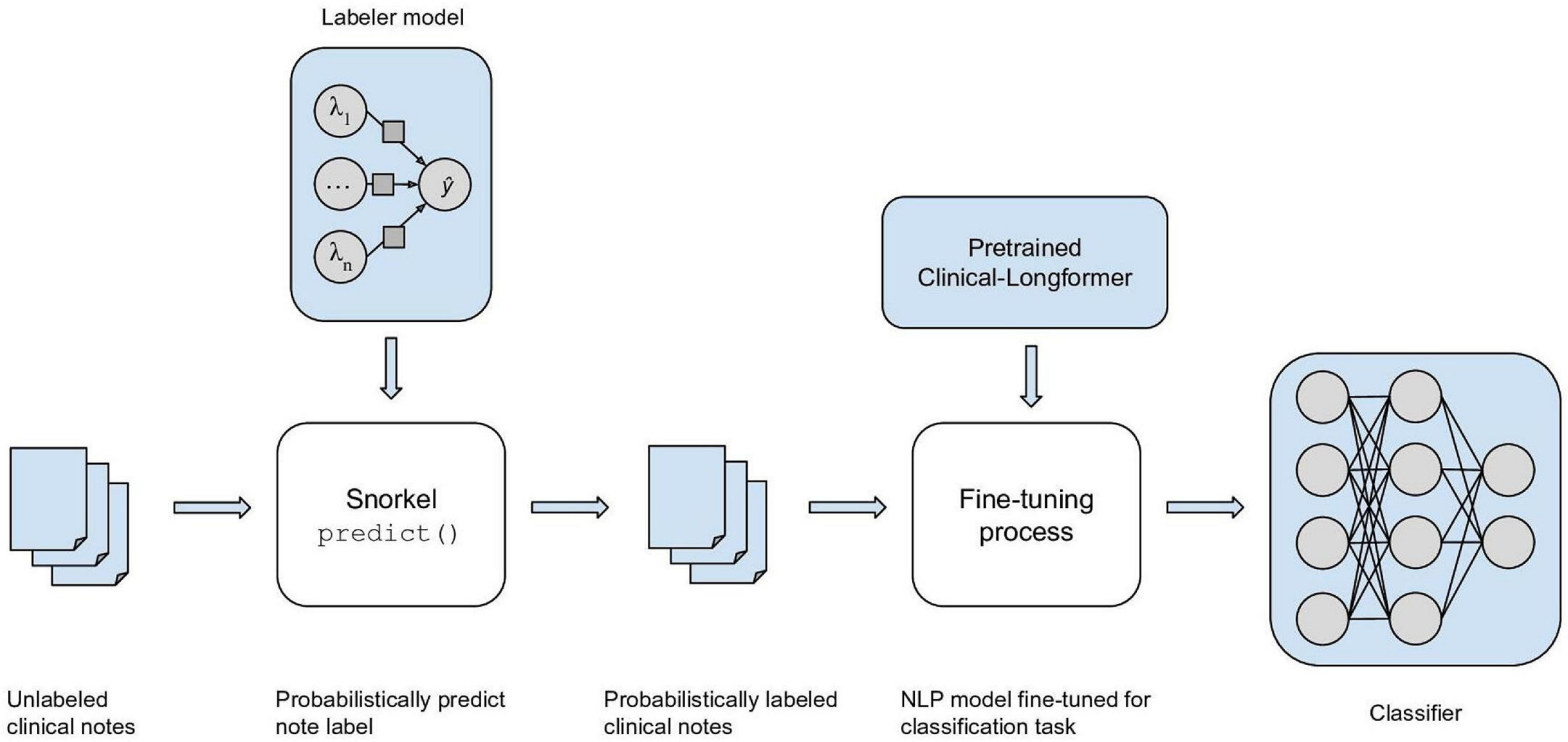
Classifier generation process. The labeler model was used to probabilistically assign labels for a large number of unlabeled clinical notes, which were then used to fine-tune a classifier to detect whether a patient received an AF prediagnosis from a wearable device. AF: atrial fibrillation; NLP: natural language processing.

Notes that were probabilistically labeled by the labeler model were then used to fine-tune a large, NLP-based classifier: Clinical-Longformer [12] (Figure 2). The resulting classifier takes plain note text as the input and classifies the note as positive (ie, includes mention of a patient receiving an AF notification, or patient-initiated cardiac testing or electrocardiogram resulting in an AF prediagnosis) or negative. When a classifier is tuned on the labeler model output, it enables generalizing beyond the labeling heuristics encoded in the labeling functions, such that the classifier can recognize more patterns.

Specifically, we fine-tuned the pretrained Clinical-Longformer for the sequence classification task, with varying training set sizes. For a single fine-tuning run, we chose the snapshot with the best *F*_1_-score on the test set as the representative. The Adam optimizer was used, with the learning rate ramping up to 1 × 10^−5^ followed by linear decay over 3 epochs. Clinical-Longformer has a maximum input length of 4096 subword tokens: 94% (53,509/56,924) of our notes fit this criterion, and notes with more tokens were trimmed. Fine-tuning other NLP-based classifiers (eg, ClinicalBERT [11], which takes a smaller number of input tokens [512 or fewer]) resulted in abysmal performance numbers (*F*_1_-score=0.21), hinting that they could not be properly fine-tuned on our lengthy clinical notes.

The test set was never presented to the classifier during the fine-tuning process. Since our data set was highly skewed toward negative samples, we stratified the training set to maintain a 1:2 ratio between the positive and negative notes. All samples were chosen randomly.

The classifier with the best *F*_1_-score was then run across the entire set of 56,924 clinical notes to identify all incidents of AF prediagnoses.

### Retrospective Cohort Study

Using the classifier, we identified patients who received an AF prediagnosis and performed 3 retrospective cohort studies comparing the characteristics of patients who received a prediagnosis to those who did not, using the same STARR data set.

First, we considered all the patients in the cohort regardless of their prior AF diagnosis. We compared the demographics, CHA_2_DS_2_-VASc (congestive heart failure, hypertension, age ≥75 years, diabetes, stroke, vascular disease, age 65-74 years, sex category) [19] score, and its related comorbidities on the date the index note was created. We defined the oldest note with a prediagnosis as the index note since it was the most likely to drive downstream medical intervention. When a patient had not received any prediagnosis, the oldest note with mention of a wearable was chosen as the index.

Second, we focused on patients who did not have a prior AF diagnosis. A patient was filtered out if the patient had received an AF diagnosis, defined as an ambulatory or inpatient encounter with SNOMED code 313217 and its descendants, prior to the index note. We then compared the same demographics and comorbidities between those who received a prediagnosis and those who did not, on the date the index note was created.

Lastly, we further confined the analysis to patients who received a clinician-assigned AF diagnosis within 60 days from the index note. Same as before, we excluded patients who had a prior AF diagnosis before the index note. Patients were then grouped based on whether they had received an AF prediagnosis from a wearable and characterized on the date they received the index AF diagnosis. In addition to the demographics and comorbidities, we also compared anticoagulant medication (Textbox 4), rhythm management medication (Textbox 5), and cardioversion rates between the 2 groups. Only the index prescription and procedure that took place within 60 days from the index diagnosis were considered.

Textbox 4. **Anticoagulant medications analyzed in this study.**

Warfarin

**Direct oral anticoagulants**

- Apixaban, dabigatran, rivaroxaban, edoxaban, and betrixaban

Textbox 5. **Rhythm management medications analyzed in this study.**

**Class I antiarrhythmics**

- Propafenone, disopyramide, quinidine, mexiletine, and flecainide

**Class II antiarrhythmics**

- Metoprolol, carvedilol, labetalol, nadolol, propranolol, carteolol, penbutolol, pindolol, atenolol, betaxolol, bisoprolol, esmolol, nebivolol, and timolol

**Class III antiarrhythmics**

- Sotalol and dofetilide

**Class IV antiarrhythmics**

- Verapamil, diltiazem, nicardipine, amlodipine, felodipine, nifedipine, isradipine, and nisoldipine

**Others**

- Digoxin

### Statistical Analysis

When compiling patient race and ethnicity information, we used the 5 categories of race defined by the US Census and denoted Hispanic as a dedicated ethnicity. A total of 11.12% (2371/21,327) of the patients were missing race and ethnicity information, so we categorized them as belonging to the *undisclosed* category.

For hypothesis testing, we used the 1-tailed Welch *t* test for continuous variables and *χ*^2^ test for categorical variables. One-tailed tests were chosen over 2-tailed tests since clinical contexts helped establish the comparison direction, providing for a stricter analysis. Statistical analysis was performed using *Pandas* [20] 1.3.0 and *SciPy* [21] 1.7.0, running on Python 3.9.6 configured through Conda 4.5.11.

### Ethical Considerations

The STARR data set is derived from consented patients only. Patients were not compensated for participation. Data analyzed in this study were not deidentified, but its analysis was conducted in a HIPAA (Health Insurance Portability and Accountability Act)–compliant, high-security environment. The Stanford University Institutional Review Board approved this study (62865).

## Results

### Labeler Model Performance

In total, 8 labeling functions were developed. Most (7/8, 88%) labeling functions used the grammatical information present in the metadata, whereas 1 (12%) used a simple dictionary-based lookup. Table 1 provides the performance of each labeling function, followed by the combined labeler model.

Since each labeling function was geared toward identifying positive samples that follow a specific pattern, each labeling function exhibited substantially higher precision than recall. By combining these labeling functions into 1 generative labeler model, we improved recall (0.72). The high labeler model accuracy (0.92) also showed that the model correctly classified negative samples. After running the labeler model on the set of 56,924 clinical notes, 5829 notes were flagged as positive, a substantial increase from the 105 positive notes identified through manual labeling.

**Table 1.** Labeling function (LF) and labeler model performance^a^.

Function or model	Target pattern	Example	Precision^b^	Recall^c^	*F*_1_-score^d^	Accuracy^e^
LF1	Simple dictionary lookup	“AF” and “wearable” and “notification”	0.90	0.33	0.51	0.87
LF2	AF^f^+verb+prep^g^+wearable	“AF noted on wearable”	0.78	0.12	0.24	0.84
LF3	Wearable+verb+AF	“Wearable notified AF”	0.91	0.42	0.55	0.89
LF4	Verb+wearable+verb+AF	“Observed wearable showing AF”	0.85	0.14	0.29	0.85
LF5	Verb+AF+prep+wearable	“Received AF from wearable”	0.81	0.15	0.31	0.85
LF6	Verb+event+prep+wearable+AF	“Got notification from wearable of AF”	0.67	0.02	0.20	0.83
LF7	Event+prep+wearable+AF	“Notified on wearable of AF”	0.74	0.10	0.27	0.84
LF8	Wearable+subject+verb+AF	“Per wearable, patient had AF”	*0.96*	0.22	0.38	0.86
Labeler model	N/A^h^	N/A	0.84	*0.72*	*0.77*	*0.92*

a Averages taken from 10-fold cross-validation on the test set of 600 manually labeled notes. Italic numbers indicate the best observed performance for each metric.

b Precision = true positive / (true positive + false positive).

c Recall = true positive / (true positive + false negative).

d *F*_1_-score = 2 × precision × recall / (precision + recall).

e Accuracy = (true positive + true negative) / (positive + negative).

f AF: atrial fibrillation.

g Prep: preposition.

h N/A: not available.

### Classifier Performance

Here, we report the performance of the classifier that was fine-tuned using the clinical notes labeled by the labeler model. Table 2 shows the average performance of the classifier on the test set, across varying training set sizes. The training set size was capped at 15,000 to maintain the 1:2 positive-to-negative ratio (the labeler model labeled 5829 notes as positive). Regardless of the training set size, the test set was excluded from the input to the fine-tuning process.

**Table 2.** Classifier performance across varying training set sizes^a^.

Training set size	Precision^b^	Recall^c^	*F*_1_-score^d^	Accuracy^e^
600	0.37	0.68	0.48	0.73
5000	0.79	*0.85*	0.81	0.93
10,000	0.84	0.81	*0.83*	*0.94*
15,000	*0.85*	0.81	*0.83*	*0.94*

a For each training set, average values are reported across 3 runs with different random seeds. For each run, the classifier snapshot with highest *F*_1_-score was used. Italic numbers indicate the best performance observed for each metric.

b Precision = true positive / (true positive + false positive).

c Recall = true positive / (true positive + false negative).

d *F*_1_-score = 2 × precision × recall / (precision + recall).

e Accuracy = (true positive + true negative) / (positive + negative).

Table 2 demonstrates how classifier performance benefits from the weakly supervised approach. In particular, a training set size of 600 emulated the hypothetical scenario where the size of the training set is limited due to manual labeling overhead. Such a small data set was not enough to adequately fine-tune Clinical-Longformer (*F*_1_-score=0.48).

As the training set size increased, the classifier obtained better performance, reaching the best average *F*_1_-score of 0.83. When compared to the labeler model in Table 1 (recall=0.72), the classifier significantly improved recall (0.81), demonstrating that the classifier managed to generalize beyond the rules specified by the labeling functions.

**Figure 3.**
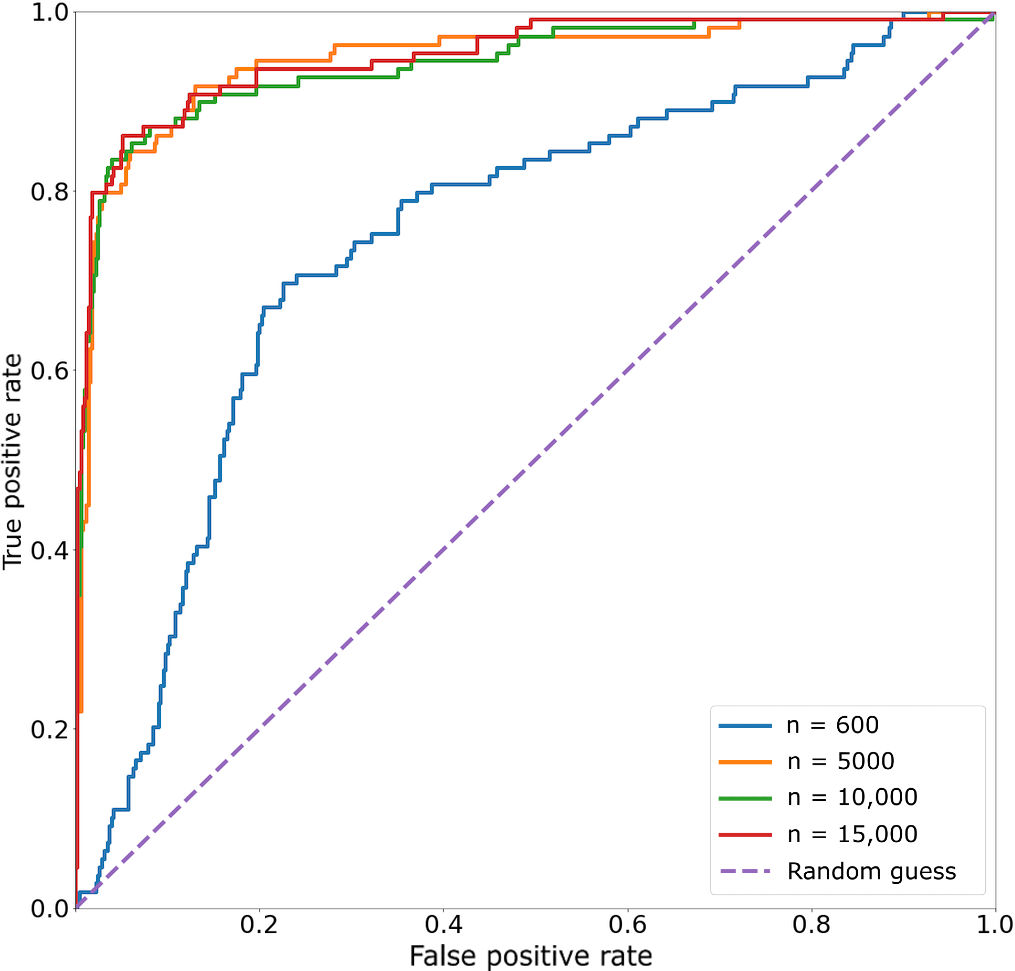
Classifier receiver operating characteristic (ROC) curve across varying training set sizes. For each training set, the best-performing (by *F*_1_-score) run was chosen among 3 runs with different random seeds. For each run, the best-performing classifier snapshot was chosen.

Figures3  4 show the comparisons of the best-performing (by *F*_1_-score) classifiers from each training set size.

**Figure 4.**
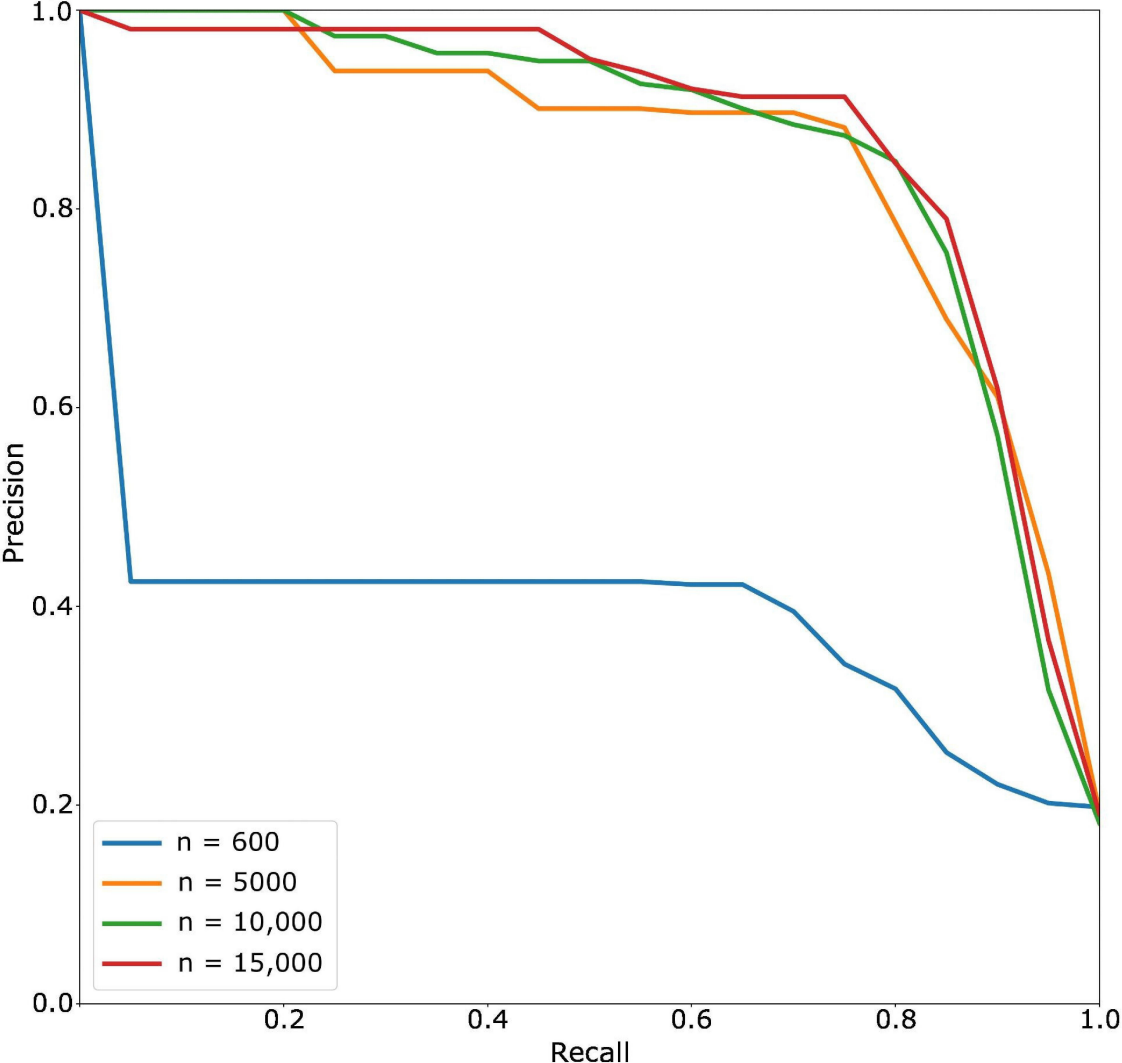
Classifier precision-recall curve across varying training set sizes. For each training set, the best-performing (by *F*_1_-score) run was chosen among 3 runs with different random seeds. For each run, the best-performing classifier snapshot was chosen.

The receiver operating characteristic curve (Figure 3) shows that even the best classifier with a training set size of 600 performed worse than classifiers from larger data set sizes. In the precision-recall curve (Figure 4), the classifier lost substantial precision for small gains in recall, further hinting that the classifier was not properly trained.

Across all training set sizes and runs, the best-performing classifier achieved an *F*_1_-score of 0.85 (accuracy=0.95). Running this classifier on 56,924 clinical notes identified 6515 notes as containing an AF prediagnosis across 2279 unique patients.

### Cohort Study: All Patients

Table 3 summarizes the characteristics of the entire cohort regardless of their prior AF diagnosis, reflecting the characteristics of generic patients that used wearables. In all, 5 patients were missing sex information and were not included in the analysis.

**Table 3.** Characteristics of all patients^a^.

Characteristics			With a prediagnosis (n=2279)	Without a prediagnosis (n=19,048)	*P* value
**Demographics**					
	Age (y), mean (SD)		63.85 (14.21)	53.53 (16.70)	<.001^b^
	**Race and ethnicity, n (%)**				<.001^b^
		Asian	295 (12.94)	3143 (16.5)	
		Black	53 (2.33)	619 (3.25)	
		Hispanic	96 (4.21)	1731 (9.09)	
		White	1613 (70.78)	11,240 (59.01)	
		Others	13 (0.57)	153 (0.8)	
		Undisclosed	209 (9.17)	2162 (11.35)	
	**Sex, n (%)**				<.001^b^
		Male	1384 (60.73)	7739 (40.63)	
		Female	895 (39.27)	11,309 (59.37)	
**Comorbidities, n (%)**					
	Congestive heart failure		341 (14.96)	1434 (7.53)	<.001^b^
	Hypertension		1267 (55.59)	6796 (35.68)	<.001^b^
	Diabetes mellitus		101 (4.43)	1018 (5.34)	.07
	Vascular disease		251 (11.01)	1582 (8.31)	<.001^b^
CHA_2_DS_2_-VASc^c^ score, mean (SD)			2.12 (1.55)	1.61 (1.35)	<.001^b^

a Measured on the date of the index note.

b Statistically significant at α=.05.

c CHA_2_DS_2_-VASc: congestive heart failure, hypertension, age ≥75 years, diabetes, stroke, vascular disease, age 65-74 years, sex category.

Patients who received an AF prediagnosis from a wearable tended to be older, with more comorbidities except for diabetes mellitus. White and male individuals constituted a larger portion of patients with a prediagnosis, who also exhibited higher CHA_2_DS_2_-VASc scores.

### Cohort Study: Patients Without a Prior AF Diagnosis

Table 4 then compares the characteristics of patients who had no AF diagnosis prior to the index note, highlighting the efficacy of wearables on the undiagnosed population.

**Table 4.** Characteristics of patients without a prior atrial fibrillation diagnosis^a^.

Characteristics			With a prediagnosis (n=1037)	Without a prediagnosis (n=16,560)	*P* value
**Demographics**					
	Age (y), mean (SD)		60.16 (15.65)	51.54 (16.28)	<.001^b^
	**Race and ethnicity, n (%)**				<.001^b^
		Asian	127 (12.25)	2890 (17.45)	
		Black	28 (2.7)	553 (3.34)	
		Hispanic	55 (5.3)	1598 (9.65)	
		White	723 (69.72)	9414 (56.85)	
		Others	3 (0.29)	136 (0.82)	
		Undisclosed	101 (9.74)	1969 (11.89)	
	**Sex, n (%)**				<.001^b^
		Male	595 (57.38)	6241 (37.69)	
		Female	442 (42.62)	10,319 (62.31)	
**Comorbidities, n (%)**					
	Congestive heart failure		85 (8.2)	696 (4.2)	<.001^b^
	Hypertension		461 (44.46)	5082 (30.69)	<.001^b^
	Diabetes mellitus		42 (4.05)	805 (4.86)	.27
	Vascular disease		95 (9.16)	1090 (6.58)	.002^b^
CHA_2_DS_2_-VASc^c^ score, mean (SD)			1.78 (1.44)	1.46 (1.23)	<.001^b^

a Measured on the date of the index note.

b Statistically significant at α=.05.

c CHA_2_DS_2_-VASc: congestive heart failure, hypertension, age ≥75 years, diabetes, stroke, vascular disease, age 65-74 years, sex category.

These patients exhibited similar characteristics to the overall cohort, where those who received an AF prediagnosis tended to be older, White, and male, with more comorbidities except for diabetes mellitus. In particular, 50.63% (525/1037) of the patients who received a prediagnosis had CHA_2_DS_2_-VASc scores of 2 or higher, warranting anticoagulation therapy [22]. In contrast, among the patients without a prediagnosis, only 35.85% (5936/16,560) had CHA_2_DS_2_-VASc scores of 2 or higher.

### Cohort Study: Patients With a Clinician-Assigned AF Diagnosis

Among those patients who did not have a prior AF diagnosis, 29.41% (305/1037) of the patients with a wearable-assigned prediagnosis received a clinician-assigned AF diagnosis within 60 days from the index prediagnosis. The average duration from prediagnosis to diagnosis was 4.74 days. In contrast, only 1.58% (262/16,560) of those patients without a prediagnosis received a clinician-assigned AF diagnosis.

Table 5 compares the clinical characteristics of those patients who received an AF diagnosis, based on whether they had received a wearable-assigned prediagnosis prior to the diagnosis.

None of the patient characteristics reported in Table 5 differed significantly between those with an AF prediagnosis and those without (all *P*>.05). However, anticoagulant prescriptions differed based on AF prediagnoses, where more patients with a prediagnosis were prescribed apixaban and rivaroxaban.

**Table 5.** Characteristics of patients with a clinician-assigned atrial fibrillation diagnosis^a^.

Characteristics				With a prediagnosis (n=305)	Without a prediagnosis (n=262)	*P* value
**Demographics**						
	Age (y), mean (SD)			64.45 (14.16)	63.65 (14.29)	.75
	**Race and ethnicity, n (%)**					.21
		Asian		35 (11.48)	27 (10.31)	
		Black		6 (1.97)	11 (4.20)	
		Hispanic		10 (3.28)	15 (5.73)	
		White		218 (71.48)	175 (66.79)	
		Others		1 (0.33)	5 (1.91)	
		Undisclosed		35 (11.48)	29 (11.07)	
	**Sex, n (%)**					.86
		Male		193 (63.28)	163 (62.21)	
		Female		112 (36.72)	99 (37.79)	
**Comorbidities, n (%)**						
	Congestive heart failure			14 (4.59)	21 (8.02)	.13
	Hypertension			111 (36.39)	109 (41.6)	.24
	Diabetes mellitus			10 (3.28)	4 (1.53)	.29
	Vascular disease			24 (7.87)	30 (11.45)	.19
CHA_2_DS_2_-VASc^b^ score, mean (SD)				1.76 (1.49)	1.81 (1.39)	.36
**Diagnosis subtype, n (%)**						.40
	Generic			230 (75.41)	213 (81.3)	
	Chronic			2 (0.66)	1 (0.38)	
	Paroxysmal			68 (22.3)	45 (17.18)	
	Persistent			5 (1.64)	3 (1.15)	
**Anticoagulant, n (%)**						
	Warfarin			1 (0.33)	3 (1.15)	.51
	**Direct oral anticoagulants**					
		Apixaban		76 (24.92)	39 (14.89)	.004^c^
		Rivaroxaban		29 (9.51)	10 (3.82)	.01^c^
**Rhythm management, n (%)**						
	**Class I antiarrhythmics**					
		Propafenone		7 (2.3)	2 (0.76)	.26
		Flecainide		17 (5.57)	8 (3.05)	.21
	**Class II antiarrhythmics**					
		Metoprolol		50 (16.39)	45 (17.18)	.89
		Carvedilol		1 (0.33)	3 (1.15)	.51
		Labetalol		6 (1.97)	4 (1.53)	.94
		Atenolol		3 (0.98)	5 (1.91)	.57
	**Class IV antiarrhythmics**					
		Verapamil		3 (0.98)	2 (0.76)	>.99
		Diltiazem		15 (4.92)	9 (3.44)	.51
		Amlodipine		4 (1.31)	3 (1.15)	>.99
	**Others**					
		Digoxin		3 (0.98)	1 (0.38)	.73
**Procedures, n (%)**						
	Cardioversion			30 (9.84)	14 (5.34)	.07

a Measured on the date of the index atrial fibrillation diagnosis. Medications that were not prescribed are omitted.

b CHA_2_DS_2_-VASc: congestive heart failure, hypertension, age ≥75 years, diabetes, stroke, vascular disease, age 65-74 years, sex category.

c Statistically significant at α=.05.

## Discussion

### Principal Findings

In this study, we applied a weak supervision–based approach to demonstrate the feasibility and efficacy of an EHR-based postmarket surveillance system for consumer wearables that render AF prediagnoses.

We first derived a labeler model from labeling heuristics expressed as labeling functions, which showed high accuracy (0.92; *F*_1_-score=0.77) on the test set. We then fine-tuned a classifier on labeler model output, to accurately identify AF prediagnoses (0.95; *F*_1_-score=0.83).

Further, using the classifier output, we identified patients who received an AF prediagnosis from a wearable and conducted a retrospective analysis to compare the baseline characteristics and subsequent clinical treatment of these patients against those who did not receive a prediagnosis.

Across the entire cohort, patients with a prediagnosis were older with more comorbidities. The race and sex composition of these patients also differed from those who did not receive a prediagnosis (*P*<.001).

Focusing on the subgroup of patients without a prior AF diagnosis (Table 4), we observed that a higher percentage of patients (525/1037, 50.63% vs 5936/16,560, 35.85%) who received a wearable-assigned prediagnosis exhibited CHA_2_DS_2_-VASc scores that warranted a recommendation for anticoagulation therapy [22]. This increased likelihood for anticoagulation therapy could be attributed to an early prediagnosis from the wearable.

In the same subgroup, patients who received a prediagnosis were 18.61 times more likely to receive a clinician-assigned AF diagnosis than those who did not. The existence of a prediagnosis was not correlated with patient demographics, comorbidities, or AF subtype at the index diagnosis (Table 5) but did correlate with anticoagulant prescription, where patients with an AF prediagnosis were more frequently prescribed apixaban (*P*=.004) and rivaroxaban (*P*=.01).

### Comparison With Prior Work

Given that more consumer wearables will be introduced with increasing prediagnostic capabilities, a surveillance framework for wearable devices is urgently needed to properly assess their impact on downstream health care [3,4]. However, publications sponsored by wearable vendors focused mostly on ascertaining the accuracy of the prediagnostic algorithm itself [1,2].

On the other hand, publications that sought to conduct postmarket surveillance relied solely on manual chart review [3,4], which is hard to scale. In a prior study on wearable notifications, clinician review of 534 clinical notes yielded only 41 patients with an AF prediagnosis [3]. With a weakly supervised approach, our clinician review of 600 notes (ie, the test set) allowed the subsequent identification of 2279 patients with a prediagnosis.

Such an improvement in recall enhanced the statistical power of our analysis. First, our cohort study findings that showed patients with an AF prediagnosis tended to be older, male, and White with higher CHA_2_DS_2_-VASc scores matches the key findings of the Apple Heart Study [1], which enrolled a much larger number of participants (n=419,297). Second, we were able to make a novel discovery in that a wearable-assigned prediagnosis increases the likelihood of patients receiving anticoagulation therapy and an eventual AF diagnosis, and we identified statistically meaningful anticoagulant prescription differences.

Prior work has applied various methods of weakly supervised learning to some form of medical surveillance [16,17,23-25]. Most relevantly, Callahan et al [23] implemented a surveillance framework for hip implants, and Sanyal et al [25] implemented one for insulin pumps. To the best of our knowledge, however, our work is the first to apply a weakly supervised approach to consumer wearable surveillance. Without prescription records, consumer wearable surveillance can be challenging to scale.

### Limitations

We acknowledge that the STARR data set is confined to a small health care system in a single geographic region, which is known [13] to serve populations with higher percentages of male, White, and older individuals. We recommend other institutions to monitor their patient population by developing their own surveillance framework using our weakly supervised methodology. In fact, work is already underway to adapt this approach for use at Palo Alto Veterans Affairs.

We could not establish causality between prediagnoses and patient characteristics. The fact that patients who are older, with more comorbidities; White; and male had a higher likelihood of receiving an AF prediagnosis may very well reflect that they are health conscious and use wearables more frequently.

### Conclusions

By providing prediagnoses, consumer wearables have the potential to affect subsequent diagnoses and downstream health care. Postmarket surveillance of wearables is necessary to understand the impact but is hindered by the lack of codified terms in EHRs to capture wearable use. By applying a weakly supervised methodology to efficiently identify wearable-assigned AF prediagnoses from clinical notes, we demonstrate that such a surveillance system could be built.

The cohort study conducted using the constructed system carried enough statistical power to verify the key findings of the Apple Heart Study, which enrolled a much larger number of patients, where patients who received a prediagnosis tended to be older, male, and White with higher CHA_2_DS_2_-VASc scores. We also made a novel discovery in that a prediagnosis from a wearable increases the likelihood for anticoagulant prescription and an eventual AF diagnosis. At the index diagnosis, the existence of a prediagnosis from a wearable did not distinguish patients based on clinical characteristics but did correlate with anticoagulant prescription.

Our work establishes the feasibility and efficacy of an EHR-based surveillance system for consumer wearable devices. Further work is necessary to generalize these findings for patient populations at other sites.

## Supplementary material

10.2196/51171 Multimedia Appendix 1 Labeling guideline developed as part of the test set generation.

## Abbreviations

AF: atrial fibrillation
CHA_2_DS_2_-VASc: congestive heart failure, hypertension, age ≥75 years, diabetes, stroke, vascular disease, age 65-74 years, sex category
EHR: electronic health record
FDA: Food and Drug Administration
HIPAA: Health Insurance Portability and Accountability Act
NLP: natural language processing
STARR: Stanford Medicine Research Data Repository
